# A pioneer Australian case of Savi-Scout™ assisted resection for breast cancer recurrence

**DOI:** 10.1093/jscr/rjac418

**Published:** 2022-09-20

**Authors:** Kristy Patricia Mansour, Chantel Thornton

**Affiliations:** Melbourne Health Department of General Surgery, Melbourne, Victoria, Australia; Epworth Richmond Department of General Surgery, Breast Surgery, Richmond, Victoria, Australia; Specialist Breast Cancer Surgery, Richmond, Victoria, Australia; Melbourne Health Department of General Surgery, Melbourne, Victoria, Australia; Epworth Richmond Department of General Surgery, Breast Surgery, Richmond, Victoria, Australia; Specialist Breast Cancer Surgery, Richmond, Victoria, Australia

## Abstract

This case describes a 48-year-old female who the first patient in Australia treated surgically with Savi-Scout™ assisted breast cancer localization, utilizing electromagnetic wave signalling for accurate depth guidance. After initial breast cancer diagnosis at age 44 treated with bilateral mastectomies and DIEP flap reconstructions, clinical surveillance found recurrent right chest wall disease. US and MRI identified a 4–6 mm interpectoral lesion; poorly differentiated metastatic micropapillary carcinoma on core biopsy. Savi-Scout™ was selected to assist localization and removal of the lesion due it’s technically challenging location. Informed consent was gained and one month pre-operatively a 12× 1.6 mm electromagnetic wave Savi-Scout™ reflector was inserted via US-guidance. A Savi-Scout™ probe guided marking, incision and dissection of subcutaneous tissues and pectoralis muscles, through localization to the reflector. The lesion and reflection were excised and confirmed on specimen radiograph, with clear histopathology margins. This technology has potential applications for challenging breast cancer cases.

## INTRODUCTION

This case report relates to a 48-year-old female who the first patient in Australia to be treated surgically with Savi-Scout™ assisted breast cancer localization. Our patient was initially diagnosed with breast cancer at age 44, treated with bilateral mastectomies and deep inferior epigastric perforator flap reconstructions. The patient had no comorbidities and a family history of mother diagnosed with breast cancer at age 58. Right chest wall recurrent disease was identified on annual post-operative clinical surveillance and ultrasound imaging.

Ultrasound guided right chest wall core biopsy and clip placement identified poorly differentiated metastatic micropapillary carcinoma. Staging investigation with computed tomography (CT) brain, chest, abdomen and pelvis was undertaken, finding no metastatic disease. Magnetic resonance imaging (MRI) identified a 4–6 mm interpectoral lesion with clip in situ, no other significant local or axillary disease, and no brachial plexus involvement. Positron emission tomography (PET) found no FDG-avid recurrent or metastatic disease and the right nodule deep to pectoralis major was not PET avid. Savi-Scout™ was selected to assist in localization and removal of the lesion due this technically challenging location of recurrence. Informed consent was gained to utilise Savi-Scout™ localization.

**Figure 1:**
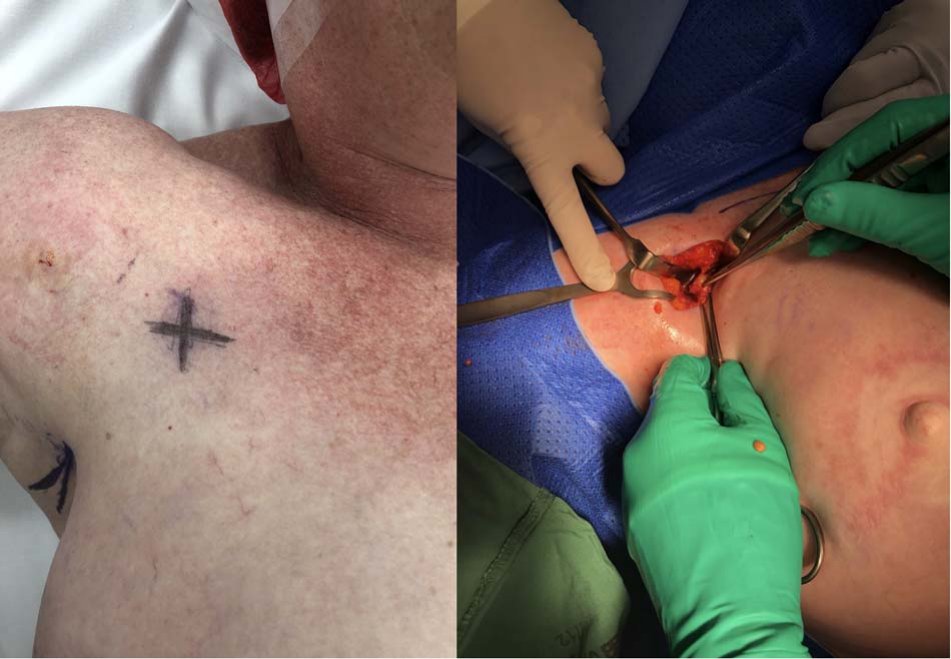
Pre-operative marking and intraoperative photograph.

**Figure 2:**
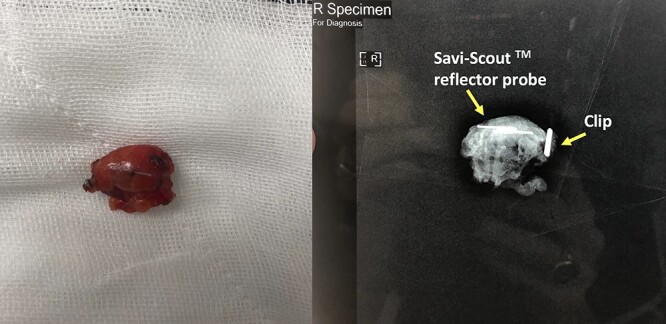
Biopsied lesion and specimen XR containing marking clips and Savi-Scout Reflector.

Pre-operative insertion of Savi-Scout™ Reflector was arranged one month prior to operation under ultrasound guidance. Simulation training using the Savi-Scout™ probe prior to operation was commenced. Pre-operatively Savi-Scout™ probe guidance was utilized to guide marking. An incision was made superiorly over the right pectoralis muscle. Dissection was made through the subcutaneous tissues and pectoralis muscles guided by Savi-Scout™ localization to the reflector node (Fig. 1). This lesion and probe were excised and confirmed on specimen radiograph (Fig. 2).

Histopathology demonstrated soft tissue containing 8 mm metastatic, poorly differentiated micropapillary breast carcinoma, clear of resection margins, oestrogen receptor positive, progesterone receptor negative and HER2 positive. These results were discussed in a multidisciplinary meeting, which recommended ongoing treatment with radiotherapy, an aromatase inhibitor and Herceptin.

## DISCUSSION

There has been an increased requirement for pre-operative localization to facilitate targeted surgical resection for non-palpable breast cancer lesions since breast screening with surveillance mammography and MRI was implemented [1, 2]. Hook wire localization was the first localization technique, providing a relatively inexpensive and widely implemented procedure, that is limited by risks of wire transection, flaying and migration [2]. Risk of migration restricts HWL to occur within 24 hours prior to operation, which can induce scheduling conflict and heightened patient anxiety due to successive procedures [3]. Radioactive seed localization (RSL) subsequently offered a wireless localization alternative for intra-operative detection using handheld gamma probe [4]. RSL is limited by requiring short duration from placement time to surgery, always requiring removal to avoid excess radiation damage to surrounding tissue, and exposing radioactive material risk to staff and patient [2]. New techniques of radiofrequency identification, magnetic and infrared localization devices are advantageous because they can be inserted at the time of initial pre-operative biopsy [2].

Savi-Scout™ localization involves pre-operative insertion of a 12× 1.6 mm electromagnetic wave reflector under radiographic guidance with US or MMG using a sterile 16 gauge introducer needle system [5]. The reflector is activated intraoperatively by infrared light from the console probe to reflect an electromagnetic wave signal back to the detection probe. This provides proximity information to continually guide surgical excision depth, providing accurate depth detection up to 6 cm from the skin surface [5].

In a systematic review of Savi-Scout localization using pooled analysis of 842 cases in 2020, an overall successful insertion/localization rate of 99.64% and retrieval rate of 99.64% was found [2]. This study also compared 263 Savi-Scout cases with 545 WGL cases finding a statistically significant difference in re-excision rate of 12.9% compared with 21.1% for WGL [2].

Accordingly, Savi-Scout offers a safe alternative to WGL with multiple benefits including minimal reflector migration, in contrast to the recognised limitation of wire migration with WGL [5]. Furthermore, Savi-Scout implementation prior to neoadjuvant therapy, at the time of percutaneous biopsy of axillary lymph nodes (PBN), has been found to facilitate 100% PBN excision following neoadjuvant therapy, compared with 75.8% PBN excision rate with conventional sentinel lymph node biopsy with technetium 99 and intraoperative isosulfan blue dye [6].

Patient satisfaction assessment using survey analysis for Savi-Scout identified that 71% of patients reported high satisfaction, and 97% of patients reported they would recommend Savi-Scout, whilst physicians reported overall patient experience at 4.1/5 [7]. Although Savi-Scout has been implemented internationally as a highly successful localization technique for non-palpable breast cancer, this report details the first application of Savi-Scout in Australia. This case highlights potential to apply this new technology for breast cancer cases where disease is difficult to localise and resect, whilst avoiding many of the inherent limitations of HWL and RSL.

## CONFLICT OF INTEREST STATEMENT

None declared.
